# Stability of the Microbiome of the Sponge *Mycale* (*Oxymycale*) *acerata* in the Western Antarctic Peninsula

**DOI:** 10.3389/fmicb.2022.827863

**Published:** 2022-04-04

**Authors:** Lea Happel, Rodolfo Rondon, Alejandro Font, Marcelo González-Aravena, César A. Cárdenas

**Affiliations:** ^1^IMBRSea International Masters Program, Ghent University, Ghent, Belgium; ^2^Helmholtz Centre for Polar and Marine Research, Alfred Wegener Institute, Bremerhaven, Germany; ^3^Departamento Científico, Instituto Antártico Chileno, Punta Arenas, Chile; ^4^Millennium Institute Biodiversity of Antarctic and Subantarctic Ecosystems (BASE), Santiago, Chile

**Keywords:** porifera, cold-water sponges, Antarctica, sponge holobiont, core community, spatial gradients

## Abstract

The sponge microbiome, especially in Low Microbial Abundance (LMA) species, is expected to be influenced by the local environment; however, contrasting results exist with evidence showing that host specificity is also important, hence suggesting that the microbiome is influenced by host-specific and environmental factors. Despite sponges being important members of Southern Ocean benthic communities, their relationships with the microbial communities they host remain poorly studied. Here, we studied the spatial and temporal patterns of the microbiota associated with the ecologically important LMA sponge *M. acerata* at sites along ∼400 km of the Western Antarctic Peninsula (WAP) to assess patterns in the core and variable microbial components of the symbiont communities of this sponge species. The analyses of 31 samples revealed that the microbiome of *M. acerata* is composed of 35 prokaryotic phyla (3 Archaea, 31 Bacteria, and one unaffiliated), being mainly dominated by *Proteobacteria* with *Gammaproteobacteria* as the most dominant class. The core community was composed of six prokaryotic OTUs, with gammaproteobacterial OTU (EC94 Family), showing a mean abundance over 65% of the total abundance. Despite some differences in rare OTUs, the core community did not show clear patterns in diversity and abundance associated with specific sites/environmental conditions, confirming a low variability in community structure of this species along the WAP. The analysis at small scale (Doumer Island, Palmer Archipelago) showed no differences in space and time in the microbiome *M. acerata* collected at sites around the island, sampled in three consecutive years (2016–2018). Our results highlight the existence of a low spatial and temporal variability in the microbiome of *M. acerata*, supporting previous suggestions based on limited studies on this and other Antarctic sponges.

## Introduction

Sponges play fundamental roles as they provide permanent, heterogeneous habitats for various organisms, provide food for other organisms, and are key in bentho-pelagic coupling (carbon and silicon cycling, oxygen depletion, and nitrogen cycling) (Bell, 2008; Janussen and Downey, 2014; Costello and Chaudhary, 2017; Cárdenas and Montiel, 2017). Sponges have important associations with a range of organisms and harbor diverse communities of microorganisms, being regarded as major contributors to marine microbial diversity (Thomas et al., 2016). In terms of the sponge–microbial symbiosis, it is considered as one of the most complex holobiont in the marine environment, due to its high number of microbial species (Pita et al., 2018). The microbiome includes microorganisms that can be found in sponge hosts among different geographical locations (generalists), whereas others are found only in particular sponge species (specialists). In general, the microbiota is known to be species-specific, consisting partly of a core microbiome (OTUs present in all individuals) and a variable part (not found in all individuals or found at different relative abundances); the prevailing proportions depend on the sponge species (Webster et al., 2004; Rodríguez-Marconi et al., 2015; Pita et al., 2016; De Mares et al., 2017; Souza et al., 2017; Lo Giudice et al., 2019). Several studies have assessed the dichotomy between Low Microbial Abundance (LMA) and High Microbial Abundance (HMA) sponges (e.g., Moitinho-Silva et al., 2017). In general, LMA sponges tend to show a usually lower species richness with a smaller proportion of core symbionts (dominated by a few groups) than HMA sponges, resulting in higher variations in the microbial composition between species. However, in general, both are relatively stable over interannual fluctuations (Erwin et al., 2015).

The sponge microbiome is expected to be influenced by the local environment, especially in LMA sponges, and since they are filter-feeding organisms, they are continuously exposed to a wide range of microorganisms inhabiting the surrounding waters (Easson et al., 2020). The impacts of environmental factors on sponge–microbe relationships have been addressed by several studies carried out at different latitudes, with some studies assessing the effects at different ambient, temporal, and/or geographic scales (Anderson et al., 2010; Erwin et al., 2012, 2015; Luter et al., 2015; Easson et al., 2020), while others tested environmental stress obtaining variable results depending on the species and also on the duration and strength of the environmental alterations (Webster et al., 2008; Fan et al., 2013; Cárdenas et al., 2014; Pineda et al., 2017; Ramsby et al., 2018). Previous studies have suggested the existence of strong interactions between geographic, environmental, and host factors influencing the microbiome of some species (Easson et al., 2020; Freeman et al., 2021). Taking into account current environmental changes occurring around the globe, it is important to better understand the sponge–microbiota patterns in terms of temporal and spatial scales as well as intra- and interspecific variations and its key ecosystem functions in order to better understand and predict potential direct and cascading effects produced by future environmental changes.

Although an important amount of research has been carried out in tropical and temperate species, the knowledge about the dynamics between host and their symbionts, especially in extreme environments such as the Antarctic region, is still limited (Lo Giudice et al., 2019), despite the fact that sponges are being considered dominant members of Antarctic benthic ecosystems (McClintock et al., 2005; Janussen and Downey, 2014; Rodríguez-Marconi et al., 2015; Cárdenas et al., 2018a; Lo Giudice et al., 2019). Existing knowledge suggests the existence of a sponge–microbiota that is host-specific, more diverse, and markedly different from those microorganisms from the surrounding seawater (Webster et al., 2004; Rodríguez-Marconi et al., 2015; Cristi et al., 2022), with Proteobacteria (mainly Alpha- and Gammaproteobacteria) as the most abundant microbial taxa, followed by Bacteroidetes. Additionally, Actinobacteria and Firmicutes have also been reported in some species (Webster et al., 2004; Rodríguez-Marconi et al., 2015; Lo Giudice et al., 2019; Steinert et al., 2019; Rondon et al., 2020). Microbial symbionts in sponges may confer a number of benefits to their host including nutrition, waste metabolism, biogeochemical and nutrient cycling, production of compounds to combat fouling and predation, and biotechnological production of pharmaceutically active secondary metabolites (Ruocco et al., 2021; Cristi et al., 2022).

The sponge *Mycale* (*Oxymycale*) *acerata* (Kirkpatrick, 1908) is one of the most dominant members of shallow-water Antarctic benthos (Cerrano et al., 1974; McClintock et al., 2005; Cárdenas et al., 2016, 2018a); however, while several studies have focused on different aspects of this species (Cerrano et al., 1974; Dayton et al., 1974; Riesgo et al., 2015; Morley et al., 2016), there is still limited information on the microbial communities associated with this ecologically important species. Current knowledge about microbial communities associated with *M. acerata* is limited to a few specimens from East Antarctica (Webster et al., 2004) and the Ross Sea Region (Papale et al., 2020; Ruocco et al., 2021), while information on *M. acerata* from the Western Antarctic Peninsula (WAP) is also limited to some samples from King George Island, Doumer Island (Cárdenas et al., 2018a,^2019^), and Deception Island (Sacristán-Soriano et al., 2020). Results from these studies show the existence of diverse microbial communities, dominated by a few OTUs. A previous study by Cárdenas et al. (2018a), reported some degree of variability between the samples of *M. acerata* from Fildes Bay, King George Island (62°S) and South Bay, Doumer Island (64°S), raising the question of whether local differences might influence microbial composition as it has been suggested for *Mycale* species from other latitudes (Anderson et al., 2010). Here, we further assess the spatial patterns of the microbiota associated with *M. acerata* at sites along ∼400 km of the WAP to assess patterns in the core and variable microbial components of the symbiont communities of these ecologically important species. We also conducted intensive sampling around Doumer Island, Palmer Archipelago to assess for differences in spatial patterns at small scale and also to further monitor the temporal stability of the microbiome of *M. acerata.*

## Materials and Methods

### Sample Collection

**FIGURE 1:**
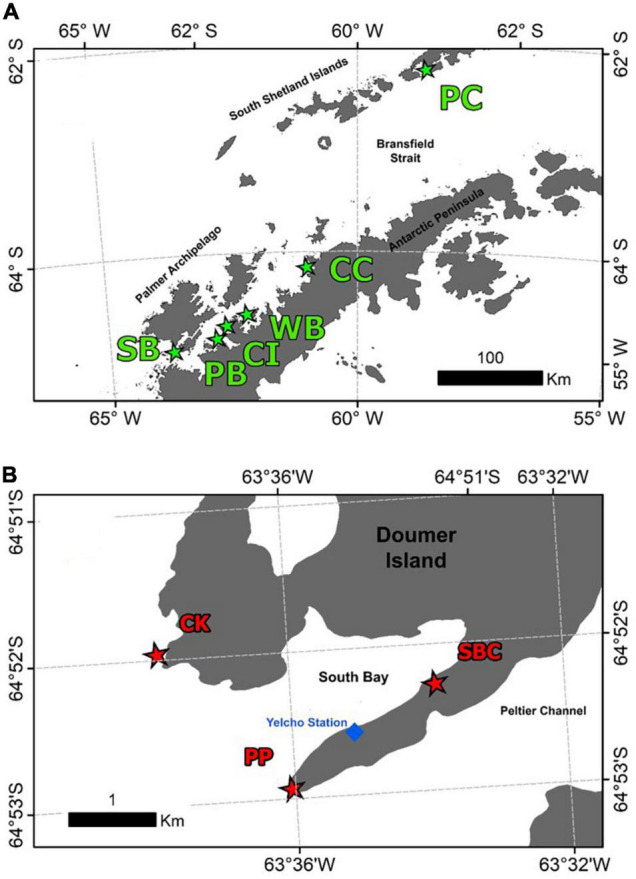
Map of the sites where samples of the Antarctic sponge *M. acerata* were collected along the WAP **(A)** and sampling was conducted at a finer scale in **(B)** South Bay (SB), Doumer Island. In grey, the Antarctic Peninsula and Islands; in white, the Southern Ocean. The sites are as follows: PC, Potter Cove; CC, Cierva Cove; WB, Wilhelmina Bay; CC, Cuverville Island; PB, Paradise Bay; SB, South Bay. Sites sampled in South Bay, Doumer Island **(B)**, SBC, South Bay coast; CK, Cape Kemp; PP, Py Point. Map constructed using Gerrish et al. (2020) High resolution vector polygons of the Antarctic coastline (7.3) (Data set). UK Polar Data Centre, Natural Environment Research Council, UK Research and Innovation (https://doi.org/10.5285/0a6d85d7-fc9c-4d68-a58d-e792f68ae9f4).

Samples of the Antarctic sponge *Mycale* (*Oxymycale*) *acerata* were collected by SCUBA diving at six areas around the WAP (Figure 1) from 2016 to 2019 (*n* = 31). Sampled sites along the WAP were carried out during the 2019 Binational Expedition to the Antarctic Peninsula, organised by National Geographic Pristine Seas Chile-Argentina in support of the Domain 1 Marine Protected Area proposal (D1MPA), currently under discussion in the Commission for the Conservation of Antarctic Marine Living Resources (CCAMLR). A more intensive sampling was carried out at Doumer Island (64°52″32″S; 63°35″02″W), Palmer Archipelago (WAP), during the Austral summer between 2016 and 2018, as part of a project studying the effect of climate change on Antarctic sponges, in order to assess the temporal and (small-scale) spatial patterns from specimens collected at sites around Doumer Island (Figure 1B), approximately 1.5 km apart from each other.

Sections of sponge individuals were aseptically collected and packed underwater in tagged resealable plastic bags filled with surrounding seawater and onboard stored in a cooler for the transportation to the laboratory. Back in the laboratory, samples were rinsed with sterilised seawater and small pieces were extracted with a sterile scalpel and then preserved in plastic tubes containing RNALater© and stored at 4°C for about 24 h, followed by the conservation of the samples at −20°C until their transportation to the Laboratorio de BioRecursos, Chilean Antarctic Institute (INACH) in Punta Arenas (Chile) for further processing.

### Genomic DNA Extraction and Sequencing

The genomic DNA extraction of the collected specimens, for 16S rRNA gene amplicon sequencing, was performed following the protocol of the DNeasy PowerSoil Kit (QIAGEN GmbH, Hilden, Germany, Version May 2017). For each replicate, about 0.3 g (± 0.03 g) of internal and external sponge tissue (to ensure obtaining the whole bacterial community) was homogenised using the Precellys© Evolution homogeniser (Bertin Technologies, Montigny-Le-Bretonneux, France). For the inhibitor removal technology (IRT) in the beginning of the protocol, the incubation after adding the corresponding solutions (C2 and C3) was performed. In the final step, the proposed alternative of DNA-free PCR-grade water instead of solution C6 and in exchange of 100 and 70 μl was used to release the DNA from the silica membrane. Internal control without sample was performed in order to identify any potential kit reagent contamination during the process. DNA concentration measurements were realised with a Nanoquant spectrophotometer (Tecan, Switzerland) followed by a PCR for template verification with Platinum Superfi™ DNA polymerase (Thermo Fisher Scientific, Waltham, MA, United States). Ten microliters of the samples, including one negative control, was sent to the Dalhousie University [CGEB—Integrated Microbiome Resource (IMR), Canada]^1^ for V4–V5 16S rRNA gene library preparation and sequencing. The multiplexing of the samples was performed with dual indexing approaches, and for the sequencing, an Illumina Miseq with paired-end 300 + 300 bp reads was used. PCR procedures, primers, and Illumina sequencing were conducted as described in Comeau et al. (2017). Raw sequences were deposited at NCBI as BioProject with the accession ID PRJNA783641.

### Analysis

The sequences obtained from the Illumina Miseq sequencing were analysed with the open-source software QIIME2 Bolyen et al. (2018). First, a quality control (qc) of the demultiplexed paired-end sequences in Sanger/Illumina 1.9 format (imported as Casava-1.8 single lane per sample), obtained from the gene library preparation of the 16S rRNA V4–V5 region, was conducted. Then, sequences were denoised with the dada2-plugin (Callahan et al., 2016), including a merge, cluster, and trimming of the reads. For the latter, the cutoff at the 3′-read-end was chosen based on the qc graph, resulting in the 295 nucleotides (nt) for the forward strand and 213 nt for the reverse strand; at the 5′-end, simply the primers were removed (forward strand: 20, reverse strand: 21).

Before the taxonomical alignment of the obtained OTUs, a general reference database Silva 138 classifier was generated, from Silva 138 to 99 taxonomy and sequences files, in order to identify without ambiguity the taxonomic affiliations. The classifier file generated and sequences of total OTUs resulting in the previous analyses were used to produce the taxonomic affiliation file.

OTUs assigned as Chloroplast (Cyanobacteria) and Mitochondria (Eukaryotes) were discarded from the two previously generated filtered tables. Analyses at Class and Order levels were produced with taxa having at least 100 sequences for at least one sample, to visualise the taxa compositions. The OTUs present in all individual samples were considered Core OTUs. Univariate measures of diversity [observed OTUs (Sobs), Chao1, Shannon, and Simpson] were calculated and plotted using R Studio scripts, and statistical differences were assessed applying Shapiro–Wilk tests to check Normality assumption and Kruskal–Wallis tests. A bubble plot was made with R studio Script to show abundance patterns across sites of the 35 most abundant OTUs.

Non-metric multidimensional scaling (nMDS) was performed to visualise multivariate patterns in the microbiome of *M. acerata*. Statistical differences were analysed using a permutational analysis of variance (PERMANOVA), based on Bray–Curtis matrices of double-square root transformed abundance data. Statistical differences were tested using 9,999 permutations of data using PRIMER v7 (Anderson et al., 2008).

Circos software online^2^ was used to perform co-occurrence microbiome networks at Class and OTU levels using taxa present in at least one sample, with at least 1% of relative abundance. Values were multiplied by 1,000 and rounded to perform analysis.

## Results

### Overall Pattern in the *Mycale* (*Oxymycale*) *acerata* Microbiome

**FIGURE 2:**
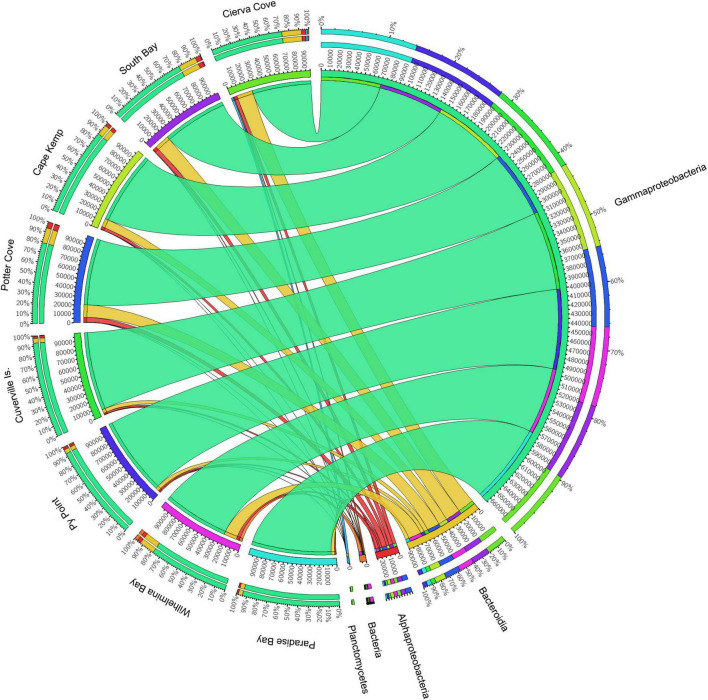
Relative abundances of microbial groups (class level) associated with the sponge *M. acerata* sampled at different sites along the WAP. The circos plot displays the relative abundance of prokaryotic classes from *M. acerata* samples collected at the different localities. The plot was generated from the OTU table grouped by class with at least 1% of relative abundance in at least one sponge sample. Values were multiplied by 1,000 and rounded without decimals in order to perform the circos plot analysis. The relative abundance of each class is directly proportional to the width of each ribbon connecting prokaryotic taxa to its respective sample. Each class is assigned a specific colour. The inner ring represents the total relative abundances (multiplied by 100) for a specific taxon and the proportion assigned to each locality. The outer ring represents the percentage of each taxon assigned to each locality.

The analysis of prokaryotic V4–V5 hypervariant region of 16s amplicon sequencing of 31 *M. acerata* samples from 8 localities yielded 1,608,433 paired-end reads. After filtering, denoising and merging sequences, 1,116,095 reads were retained and 1,056,898 mom-chimeric sequences were left for further analysis. Sequences with Chloroplast and mitochondrial affiliation were removed before performing statistical analysis keeping 1,024,506 sequences corresponding to a total of 854 OTUs recorded for *M. acerata*. The obtained OTUs belong to 34 affiliated phyla, 3 Archaea, and 31 Bacteria. Seventy-two class affiliations were found, with Bacteroidia, Alphaproteobacteria, and Gammaproteobacteria being the most representative ones. Gammaproteobacteria dominated the microbiome of all samples (Figure 2), constituting over 83% of the total abundance. In contrast, Alphaproteobacteria and Bacteroidia showed lower values, reaching about 3.2 and 11.3% of the total abundance, respectively.

### Spatial Patterns in the Microbiome of *Mycale* (*Oxymycale*) acerata

#### Alpha Diversity

**FIGURE 3:**
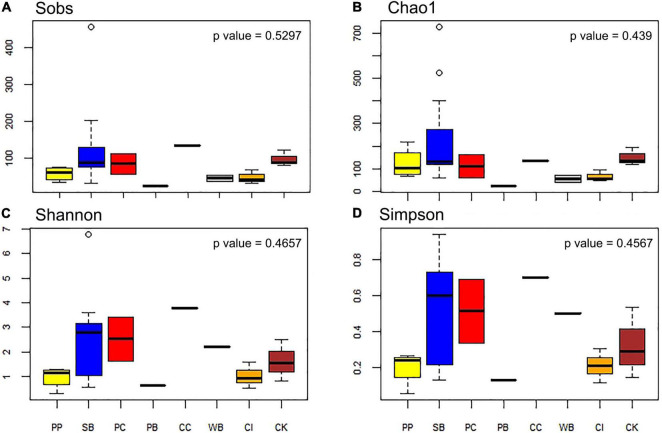
Diversity measures of microbial communities associated with the sponge *M. acerata* sampled at different sites along the WAP. **(A)** Observed OTUs, **(B)** Chao1 diversity index, **(C)** Shannon diversity index, **(D)** Simpson diversity index. PC, Potter Cove; CC, Cierva Cove; WB, Wilhelmina Bay; CI, Cuverville Island; PB, Paradise Bay; SB, South Bay; PP, Py Point; CK, Cape Kemp.

The number of OTUs ranged from 30 to 456. The mean number of OTUs was highest at 120.67 (± 104.33 SD) at South Bay and the lowest was recorded at Wilhelmina Bay with 45 ± 11.31 (Figure 3 and Table 1). The alpha diversity indices assessed in this study (Sobs, Chao1, Shannon, and Simpson) did not show significant differences between localities (*p* = 0.5), highlighting the presence of homogeneous patterns in diversity indexes across the WAP.

**TABLE 1:** Diversity measures of the microbial communities recorded in samples of *Mycale* (*Oxymycale*) *acerata* collected at sites along the WAP.

Site		Obs OTUs	Chao1	Shannon	Simpson
Potter Cove (2)	Mean	84.00	110.50	2.53	0.51
	*SD*	41.01	74.25	1.26	0.25
Cierva Cove (1)		135.00	135.00	3.79	0.70
Wilhelmina Bay (2)	Mean	45.00	53.50	2.21	0.50
	*SD*	11.31	21.92	0.02	0.00
Cuverville Island (3)	Mean	47.00	65.00	1.02	0.21
	*SD*	19.47	24.43	0.53	0.09
Paradise Bay (1)		25.00	25.00	0.65	0.13
Py Point, Doumer Island (4)	Mean	57.25	123.51	0.97	0.20
	*SD*	19.48	67.93	0.44	0.10
Bahia South, Doumer Island (15)	Mean	120.67	234.85	2.48	0.51
	*SD*	104.33	187.53	1.61	0.29
Cape Kemp, Doumer Island (3)	Mean	96.33	149.33	1.62	0.32
	*SD*	21.57	40.25	0.83	0.20

*The number of samples per site is shown in parentheses.*

#### Structure and Abundance Patterns

At Class level, only three lineages occurred in high abundance, with Gammaproteobacteria as the most dominant across all sites (83.22 ± 8.68%) followed by Bacteroidea (11.29 ± 5.86%), which showed a similar pattern across sites (Figure 2). A third class (Alphaproteobacteria) also showed consistent presence in all sites, however, showing a much lower abundance (3.23 ± 1.39%) compared with the other two classes. The analysis of the community structure at Class level showed differences among sites [PERMANOVA *F*_(7, 30)_ = 1.6921; *p* = 0.0053]; however, pairwise comparisons showed that differences existed only when comparing Cierva Cove (*n* = 1) with Cuverville Island (*n* = 3) (*p* = 0.05) with all other pairwise comparisons being not statistically significant.

**FIGURE 4:**
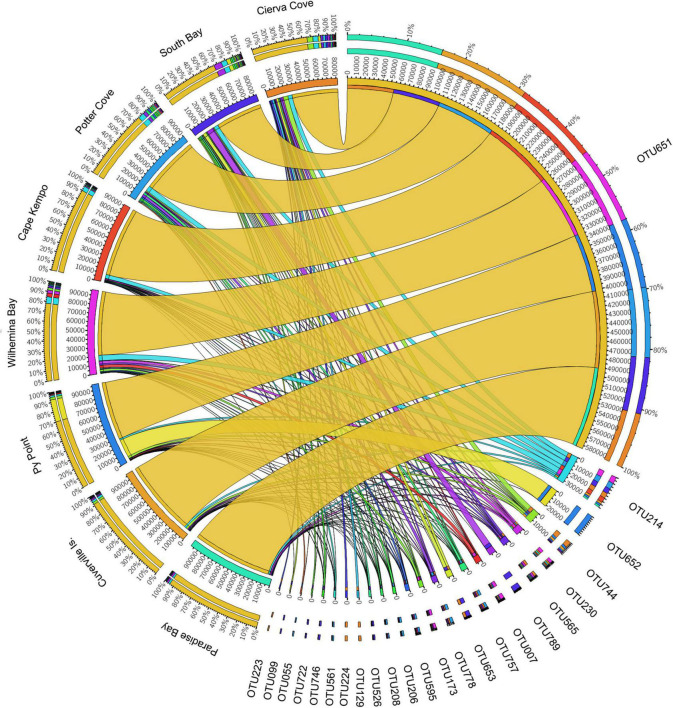
Relative abundances of microbial OTUs associated with the sponge *M. acerata* sampled at different sites along the WAP. The circos plot displays the relative abundance of most abundant prokaryotic OTUs from *M. acerata* samples collected at the different localities. The plot was generated from OTUs with at least 1% of relative abundance in at least one sponge sample. Values were multiplied by 1,000 and rounded without decimals in order to perform the circos plot analysis. The relative abundance of each OTU is directly proportional to the width of each ribbon connecting prokaryotic taxa to its respective sample. Each OTU is assigned a specific colour. The inner ring represents the total relative abundances (multiplied by 100) for a specific taxon and the proportion assigned to each locality. The outer ring represents the percentage of each taxon assigned to each locality.

**FIGURE 5:**
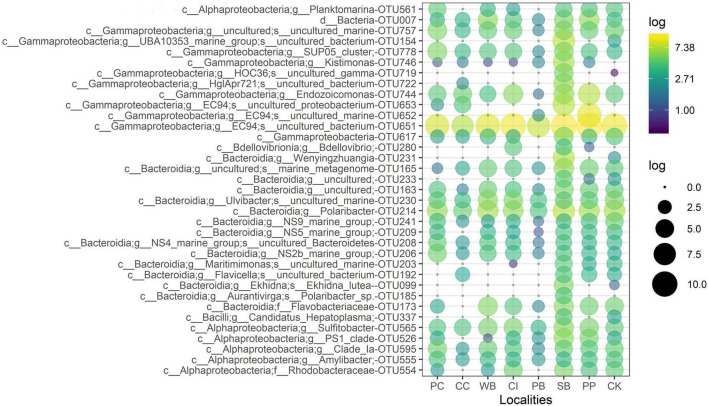
Relative abundance of the 35 most abundant microbial OTUs from *M. acerata* sampled at different sites along the WAP. The letters before affiliation represent taxonomic levels: d, Domain; c, Class; g, Genus; f, Family; and s, Species. PC, Potter Cove; CC, Cierva Cove; WB, Wilhelmina Bay; CI, Cuverville Island; PB, Paradise Bay; SB, South Bay; PP, Py Point; CK, Cape Kemp.

The OTU651 (*Uncultured bacterium*, EC94 family) was highly dominant in all sites as shown in the Circos plot (Figure 4), with a mean abundance of 72.99 (± 13.61). The OTU 214 (*Polaribacter*) was the second most important contributor; however, it was much less abundant compared with OTU651 with an average of 4.13 ± 1.83%. Several other OTUs showed a similar pattern, being consistently present in all sites; however, their relative abundance was considerably lower (Figure 5 and Supplementary File 1).

**FIGURE 6:**
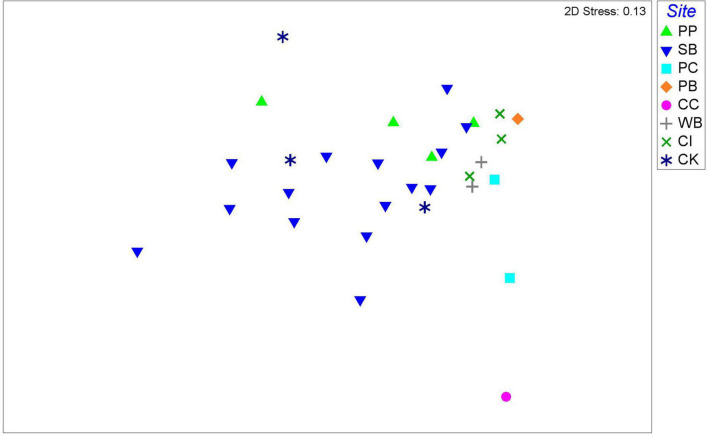
Non-metric multidimensional scaling plot of microbial communities associated with the sponge *M. acerata*, with symbols representing different sites sampled along the WAP. The analysis is based on fourth root transformed relative abundances of microbial taxa. PC, Potter Cove; CC, Cierva Cove; WB, Wilhelmina Bay; CI, Cuverville Island; PB, Paradise Bay; SB, South Bay; PP, Py Point; CK, Cape Kemp.

Overall, the nMDS does not show a clear separation of *M. acerata*-associated microbial communities between the different sites along the WAP (Figure 6). Although the PERMANOVA analysis at the OTU level detected significant differences [*F*_(7, 30)_ = 1.7274; *p* = 0.028], the pairwise analyses showed that differences existed only in comparisons between South Bay (*n* = 15) and Cierva Cove (*n* = 1) (*p* = 0.0367) and between South Bay (*n* = 15) and Cuverville Island (*n* = 3) (*p* = 0.037).

#### Core Community

Six prokaryotic OTUs were present in all *M. acerata* samples collected across the WAP, being all bacterial OTUs (Supplementary Table 1). The most abundant OTU of the core community was a gammaproteobacterial OTU (OTU651) belonging to the EC94 Family, showing a mean abundance over 65% (± 31.82 SD). The second most abundant core OTU (OTU214) corresponded to Polaribacter (Flavobacteriaceae) with a mean abundance of 4.42 ± 4.12%. The other four core OTUs showed mean relative abundances of less than 1%, mainly belonging to gammaproteobacterial OTUs and an unclassified Bacteria (Supplementary Table 1).

### Temporal Patterns and (Small Scale) Spatial Patterns

The analysis to further assess spatial patterns at a smaller scale from samples collected in consecutive years (2016–2018) did not show differences among sites sampled around Doumer Island [PERMANOVA *F*_(2, 21)_ = 1708; *p* = 0.3415]. In addition, no temporal differences [*F*_(2, 21)_ = 1869.3; *p* = 0.2661] or interactions between site and year were recorded [*F*_(2, 21)_ = 1762.5; *p* = 0.3402].

## Discussion

Previous research on sponge–microbe associations on Antarctic species has provided information on diversity and structure of associated microbial communities, with still limited data on host specificity or temporal and spatial patterns of their microbiome. Despite the ecological importance of the sponge *Mycale* (*Oxymycale*) *acerata*, few studies have provided insights into the microbiome of this species, with information collected from some sites at the WAP (Cárdenas et al., 2018a,^2019^; Sacristán-Soriano et al., 2020) and other areas of Antarctica (Webster et al., 2004; Papale et al., 2020; Ruocco et al., 2021); however, most of them were based on limited or non-replicated sampling or based on a limited number of localities. Several studies have assessed the latitudinal variation of the sponge microbiome, reporting relatively low within-host variation; however, most of them have focused on tropical or temperate species (Anderson et al., 2010; Taylor et al., 2013; Marino et al., 2017; Easson et al., 2020). The present study is the first to be published on spatial patterns of the microbiome of the Antarctic sponge *M. acerata*, based on several replicates collected at different sites spanning 400 km of the WAP. This study of a cold-water sponge species provides consistent results with what has been reported for other tropical and temperate species, reporting low spatial and temporal variability in the sponge microbiome without a strong influence from the local environment.

The microbiome of *M. acerata* was dominated by Gammaproteobacteria (over 80% of the total abundance), with Bacteroidia and Alphaproteobacteria as the second and third most important groups. Our findings are similar to results from previous studies analysing samples from single localities around the WAP (Cárdenas et al., 2018a,^2019^; Sacristán-Soriano et al., 2020; Supplementary Table 2) and also with *M. acerata* samples from Terra Nova Bay, Ross Sea; however, in the latter, the dominance of Gammaproteobacteria is not as marked as in samples from the WAP, reaching about 40% of the total abundance (Papale et al., 2020). Regarding OTU richness, values reported here fall within the range reported by previous studies of *M. acerata* samples collected at sites around the South Shetland Islands (Maritime Antarctica) and the WAP (see Supplementary Table 2). However, it is important to mention that, in some cases, values differ mainly due to the hypervariable regions analysed by the different studies, as was previously demonstrated by Cárdenas et al. (2018a) when comparing two species of *Mycale*.

In addition, in accordance with what has been described from these studies, the microbiome is strongly dominated by very few OTUs that encompass most of the total abundance. In our study, six OTUs were present across all samples with a gammaproteobacterial OTU (OTU651), EC94, a group of predominant non-culturable bacteria, previously described in *M. acerata* (Cárdenas et al., 2019) reaching over 60% of the total abundance.

The results obtained in this study show the same trend and confirm the importance that group EC94 may have in this species of cold-water sponges. Although this group has been described with highest overall abundance for temperate and tropical water sponges (Jackson et al., 2013; Gauthier et al., 2016; Steinert et al., 2016), temperature does not seem to be a driving factor in its prevalence and bacterial community stability in *M. acerata*. Recently, Taylor et al. (2021) using metagenomics in-depth phylogenetic analyses, metatranscriptomics, and fluorescence *in situ* hybridisation microscopy, proposed this as a group of bacteria as a new gammaproteobacterial order that named ^U^Tethybacterales. This order shares a heterotrophic lifestyle and is almost exclusively present in sponges, showing diverse morphologies, predicted substrate preferences, and localisation within the sponge tissue. Future studies should confirm the functional role of this group in *M. acerata* associated with their functional capacity.

Another core group was the uncultured clade SUP05; these sulphur-oxidising marine chemoautotrophs have been previously described in hydrothermal vent sponges, and they play a role in sulphur oxidation to reduce sulphur compounds as an energy source (Zhou et al., 2019), and are also involved in sequential reduction of nitrate (NO_3_^–^) to nitrogenous gases or indirectly by dissimilative NO_3_^–^ reduction to ammonia (Shah et al., 2017). SUP05 has been described as predominant members in marine environments such as hydrothermal vents, and oxygen minimum zones across the world’s oceans, constituting symbiotic associations with hydrothermal vent invertebrates (Anantharaman et al., 2013). It is not surprising that this group is present in Antarctica as it has been described from seawater samples and in the Antarctic sponge *Sphaerotylus antarcticus* (Sacristán-Soriano et al., 2020). Although previous works have predicted some potential functional roles of sponge-associated microbial communities associated to Antarctic sponges (Cárdenas et al., 2019; Steinert et al., 2019; Moreno-Pino et al., 2020; Cristi et al., 2022), new studies on metagenomics or genome sequencing studies are needed in order to confirm this and other functional roles played by associated microbial symbionts on Antarctic environments.

*Polaribacter* (OTU214), a microorganism belonging to the marine Bacteroidetes, which represent the third most abundant group of marine bacterioplankton, after cyanobacteria and proteobacteria (Gosink et al., 1998), was also an important member of the core community of *M. acerata.* This group appears to be widely distributed in marine polar environments as previous works from other areas of Antarctica have reported (Webster et al., 2004; Savoca et al., 2019; Papale et al., 2020). Some studies have also shown that this cold-adapted bacterial group is able to produce interesting exopolysaccharide molecules with some promising medical applications (Sun et al., 2015; Ruocco et al., 2021).

The few available studies on *Mycale* species around the globe have shown contrasting results. For instance, spatial variability has been reported in *Mycale hentscheli* from New Zealand coasts (Anderson et al., 2010), but studied with a different molecular technique such as DGGE; others such as *Mycale* sp. from deep waters in France seem to show little variation in its microbiome compared with other sponge species (Reveillaud et al., 2014). The slight variations observed among samples of the Antarctic sponge *M. acerata* are within what is expected considering the consistent patterns of the core and variable OTUs observed at temporal scales (Cárdenas et al., 2019) and also with specific individuals (Sacristán-Soriano et al., 2020). A recent paper (Cristi et al., 2022) studying the microbiome of three Antarctic sponge species suggests that the microbiome and host species are not equally conserved among sponge species, which may be explained by differences in the acquisition of the microbes among species. This pattern agrees with what seems to be the case for *M. acerata* samples in this study and also from what was previously recorded by Sacristán-Soriano et al. (2020) in Deception Island where one sample out of three showed a slightly different pattern with an enrichment for Thaumarchaeota; however, overall, the dominance of core OTUs was consistent among sites. Similar studies from tropical latitudes reported an absence of significant variation in the microbiome of *Cliona* species at similar geographic distances, with greater variation found only at large distance scales (>1,000 km) (Easson et al., 2020).

The physical environment around the WAP is a very complex system, strongly influenced by several features including the circulation of warmer water of the Antarctic Circumpolar Current (ACC), the cold waters from the Weddell Sea flowing along the mainland coast of the WAP, and a network of glaciers (Moffat and Meredith, 2018). Such characteristics have caused, for instance, clear patterns of glacier change along the WAP, with glaciers in the south undergoing considerable retreat compared to those in the northwest are more influenced by cooler waters (Cook et al., 2016). In addition, slightly different (absolute) temperature and annual range (Barnes et al., 2006) and higher local variability (Cárdenas et al., 2018b) can produce differences in the characteristics of the communities. For instance, differences in sea bottom temperature, influenced by the effect produced by the ACC and the Weddell Gyre, have been suggested as key drivers explaining significant differences in community structure between northern areas of the Scotia Sea and the WAP (Lockhart and Jones, 2008; Friedlander et al., 2020). The importance of environmental variability at local scales influencing physiological responses of Antarctic species has also been suggested, where species inhabiting more stable zones might show different responses than those from areas with higher variability in seawater temperature (Cárdenas et al., 2018b); however, this remains to be tested. The stability and structure of the microbiome of species such as *M. acerata* could be expected to be influenced by the local environment; however, our results do not show clear patterns associated with specific sites, and the taxonomic pattern was highly homogeneous with the same OTUs and Classes being the most abundant for the localities of WAP considered in this study. Despite the fact that our sampling was conducted across different years (2016–2019), we found consistent stability in the microbiome at both small and large spatial scales, which was supported by our analysis, when the effect of year was tested. The observed temporal stability is similar to what was reported from a single individual of this species monitored for 3 consecutive years that was not affected by sudden increases in seawater temperature, showing no signs of variation after such a high variability event (Cárdenas et al., 2019). In contrast, we found a higher degree of variation in some particular individuals, a situation that was previously reported in a comparison of samples from Deception Island (Sacristán-Soriano et al., 2020). The stability of less complex microbiomes and the capacity of the sponge holobiont to select microorganisms from the environment in its benefit under stressing conditions (Oliveira et al., 2020) could be essential in such an extreme environment. In this regard, the high stability in the microbiome of *M. acerata* together with high growth rates (Dayton et al., 1974) might play a key role for the success of this species in colonising Antarctic benthos.

## Conclusion

The present study confirms the high stability of the microbiome of the common Antarctic sponge *M. acerata* that was previously reported at temporal scales based on limited sampling (Cárdenas et al., 2016, 2019; Sacristán-Soriano et al., 2020). Our study, which is based on a more intense sampling, shows a highly consistent pattern in the core microbiome of this species, independent of the site, environmental conditions, and the geographical scale (small or large) in the WAP. The high stability in the microbiome of *M. acerata* at a range of spatial and temporal scales, along with other biological characteristics described in earlier studies, may explain its high abundance in some areas. Further studies are, however, needed as we are still making the first steps in understanding and expanding on our still limited knowledge of sponge associated prokaryotic communities in polar environments, and their benefits for the host and the Antarctic ecosystem.

## Data Availability Statement

The datasets presented in this study can be found in online repositories. The names of the repository/repositories and accession number(s) can be found in the article/Supplementary Material.

## Author Contributions

CC conceived the study and took the samples. LH processed the samples. LH and RR conducted the bioinformatics analyses. LH, RR, and CC interpreted the data. CC, RR, MG-A, and AF drafted the manuscript. All authors reviewed and approved the final manuscript.

## Conflict of Interest

The authors declare that the research was conducted in the absence of any commercial or financial relationships that could be construed as a potential conflict of interest.

## Publisher’s Note

All claims expressed in this article are solely those of the authors and do not necessarily represent those of their affiliated organizations, or those of the publisher, the editors and the reviewers. Any product that may be evaluated in this article, or claim that may be made by its manufacturer, is not guaranteed or endorsed by the publisher.

## Footnotes

1
https://imr.bio/

2
http://www.circos.ca
