# Supplementary material for: Stability of the Microbiome of the Sponge Mycale (Oxymycale) acerata in the Western Antarctic Peninsula
Source: Front Microbiol. 2022 Apr 4;13:827863. doi: 10.3389/fmicb.2022.827863 (PMC9014287; doi:10.3389/fmicb.2022.827863)
Supplement: Supplementary file 1 [file Table_1.DOCX]

**Supplementary Table 1.** Mean (± Standard Deviation) relative abundance of core community (core OTUs) associated with the sponge *M. acerata* sampled at different sites along the WAP. Core OTUs were considered when they were present in all sampling stations for all localities.

| OTU | OTU Affiliation | **Py Point** | **South Bay** | **Potter Cove** | **Paradise Bay** | **Cierva Cove** | **Wilhelmina Bay** | **Curverville Island** | **Cape Kemp** |
| --- | --- | --- | --- | --- | --- | --- | --- | --- | --- |
| OTU7 | d__Bacteria;__;__;__;__;__;__ | 1,03  ± 1,80 | 0,70  ± 0,60 | 0,22  ± 0,21 | 0,20  ± 0 | 0,96  ± 0 | 3,42  ± 1,25 | 0,39  ± 0,28 | 0,12  ± 0,17 |
| OTU163 | d__Bacteria;p__Bacteroidota;c__Bacteroidia;o__Flavobacteriales;f__Cryomorphaceae;g__uncultured;__ | 0,28  ± 0,31 | 0,67  ± 0,73 | 0,72  ± 0,36 | 0,17  ± 0 | 0,19  ± 0 | 0,90  ± 0,17 | 0,22  ± 0,09 | 0,45  ± 0,42 |
| OTU214 | d__Bacteria;p__Bacteroidota;c__Bacteroidia;o__Flavobacteriales;f__Flavobacteriaceae;g__Polaribacter;__ | 1,84  ± 1,73 | 5,18  ± 5,00 | 6,10  ± 2,62 | 1,78  ± 0 | 5,03  ± 0 | 6,45  ± 0,08 | 2,07  ± 0,69 | 4,61  ± 5,90 |
| OTU651 | d__Bacteria;p__Proteobacteria;c__Gammaproteobacteria;o__Burkholderiales;f__EC94;g__EC94;s__uncultured_bacterium | 68,19  ± 44,58 | 54,87  ± 36,29 | 68,12  ± 18,69 | 93,15  ± 0 | 53,90  ± 0 | 74,88  ± 1,30 | 88,91  ± 5,04 | 81,87  ± 12,45 |
| OTU757 | d__Bacteria;p__Proteobacteria;c__Gammaproteobacteria;o__Oceanospirillales;f__Nitrincolaceae;g__uncultured;s__uncultured_marine | 0,25  ± 0,24 | 0,78  ± 0,91 | 2,57  ± 1,98 | 0,14  ± 0 | 1,23  ± 0 | 0,87  ± 0,09 | 0,45  ± 0,25 | 0,71  ± 0,90 |
| OTU778 | d__Bacteria;p__Proteobacteria;c__Gammaproteobacteria;o__Thiomicrospirales;f__Thioglobaceae;g__SUP05_cluster;__ | 0,11  ± 0,16 | 0,74  ± 1,14 | 2,86  ± 1,81 | 0,17  ± 0 | 1,31  ± 0 | 0,65  ± 0,05 | 0,33  ± 0,14 | 0,13  ± 0,09 |
